# Ketamine and rapid-acting antidepressants: a new era in the battle against depression and suicide

**DOI:** 10.12688/f1000research.14344.1

**Published:** 2018-05-24

**Authors:** Ronald S. Duman

**Affiliations:** 1 Department of Psychiatry, Laboratory of Molecular Psychiatry, Yale University School of Medicine, New Haven, CT 06508, USA

**Keywords:** Ketamine, mTOR, antidepressants, depression, suicide

## Abstract

Therapeutic medications for the treatment of depression have serious limitations, particularly delayed onset and low rates of efficacy. However, the discovery that a single subanesthetic dose of ketamine, a glutamate NMDA receptor channel blocker, can produce a rapid (within hours) antidepressant response that is sustained (about 1 week), even in patients considered treatment-resistant, has invigorated the field. In addition to these remarkable actions, ketamine has proven effective for the treatment of suicidal ideation. Efforts are under way to develop ketamine-like drugs with fewer side effects as well as agents that act at other sites within the glutamate neurotransmitter system. This includes ketamine metabolites and stereoisomers, drugs that act as NMDA allosteric modulators or that block mGluR2/3 autoreceptors. In addition, targets that enhance glutamate neurotransmission or synaptic function (or both), which are essential for the rapid and sustained antidepressant actions of ketamine in rodent models, are being investigated; examples are the muscarinic cholinergic antagonist scopolamine and activators of mechanistic target of rapamycin complex 1 (mTORC1) signaling, which is required for the actions of ketamine. The discovery of ketamine and its unique mechanisms heralds a new era with tremendous promise for the development of novel, rapid, and efficacious antidepressant medications.

## Introduction

Major depressive disorder affects nearly 20% of the population at some point during the life span and is estimated to be the number one cause of disability by 2020 ^1– 4^. The deleterious effects of depression are compounded by the lack of fast and efficacious treatment regimens, as currently available medications are effective in only about two-thirds of patients and there is a significant therapeutic time lag of weeks to months ^5^. The efficacy and time lag limitations of current medications are particularly problematic for a patient population at elevated risk for suicide, which has increased significantly over the past 4 years ^6, 7^.

These deficiencies highlight a major unmet need for the treatment of depression and underscore the impact of new rapid-acting antidepressant agents, notably the NMDA receptor antagonist ketamine. A single subanesthetic dose of ketamine produces a therapeutic response within a few hours that lasts for about 7 days ^8, 9^. Equally surprising is that ketamine is effective in severely depressed patients who are considered treatment-resistant (that is, patients who have failed to respond to two or more typical antidepressants). In addition, ketamine has proven effective for the rapid reduction of suicide ideation ^10^. The discovery of the rapid, efficacious, and sustained effects of ketamine is arguably the greatest breakthrough in the field of depression in over 60 years since the development of the monoaminergic antidepressants in the 1950s.

Here, we will provide a brief history and overview of the discovery of ketamine and how it impacts the synaptic pathophysiology of depression and current evidence on the molecular and cellular mechanisms underlying the actions of ketamine. Then we will discuss how the ketamine discovery has stimulated the development of novel agents with fewer side effects, including drugs that act at the glutamate-NMDA receptor but also other agents that impact synapse number and function via other pathways.

## Discovery of ketamine and NMDA receptor antagonists as rapid-acting agents

Most drug development efforts in the depression field have focused on the serotonin and norepinephrine neurotransmitter systems since the development of the monoamine reuptake blocker tricyclic antidepressants. However, studies of the glutamatergic neurotransmitter system and the mechanisms underlying synaptic plasticity stimulated drug development efforts in these areas. Early studies demonstrated that typical antidepressants altered the affinity of the NMDA receptor glycine site, suggesting the possibility that decreased NMDA receptor function contributes to an antidepressant response. This hypothesis was directly tested first by Krystal, Berman, Charney, and colleagues at Yale when they investigated a single subanesthetic dose (0.5 mg/kg, intravenous [IV] infusion over the course of 40 minutes) of ketamine and found that patients started reporting improvement of depressive symptoms within a matter of a few hours ^8^. These improvements occurred after the initial psychotic and dissociative effects of ketamine, which occur in the first 60 minutes. The rapid and efficacious antidepressant actions of ketamine were confirmed in a larger double-blind, placebo-controlled study by Zarate, Charney, and colleagues at the National Institute of Mental Health ^9^ and subsequently by many other studies from a number of clinical research groups, largely erasing any doubts of the incredible antidepressant actions of ketamine ^11– 13^.

### Ketamine reverses the synaptic abnormalities caused by stress

**Figure 1.**
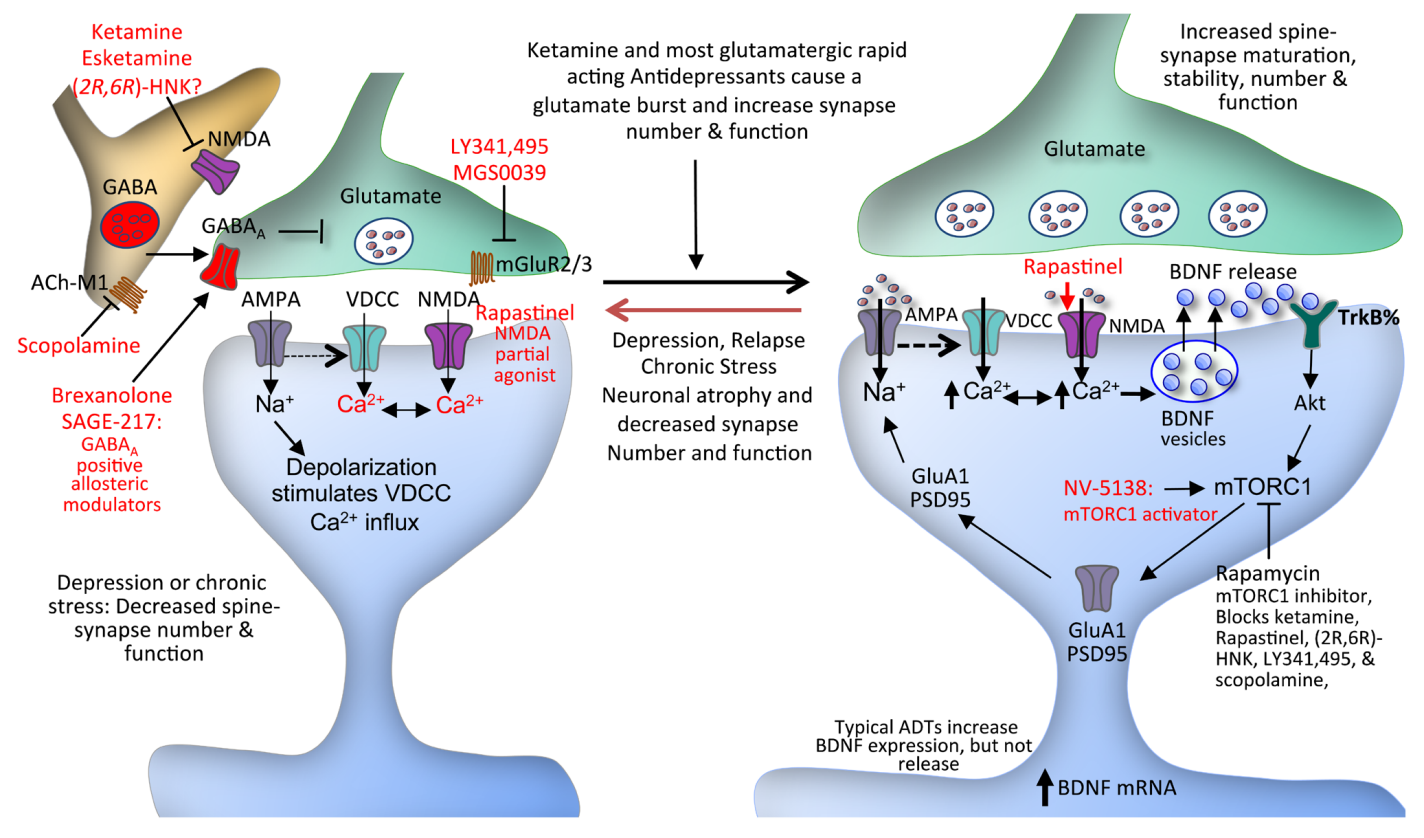
Schematic model for the initial cellular target sites of rapid-acting antidepressants and subsequent synaptic changes. Stress and depression cause neuronal atrophy and decreased synapse number in the medial prefrontal cortex (PFC) and hippocampus that is associated with the depressive symptoms and behaviors in rodent models. Conversely, fast-acting antidepressants like ketamine rapidly increase synapse number and function and reverse the synaptic deficits caused by chronic stress. The synaptic actions of ketamine, as well as several other agents (that is, esketamine, [2R,6R]-hydroxynorketamine, mGluR2/3 antagonists LY341,495 and MGS0039, and scopolamine acting at acetylcholine muscarinic 1 receptors), are activity-dependent and are thought to result from a burst of glutamate via blockade of receptors on tonic firing GABA interneurons, resulting in disinhibition of glutamate transmission. This burst of glutamate causes activity-dependent release of brain-derived neurotrophic factor (BDNF), stimulation of TrkB-Akt and mechanistic target of rapamycin complex 1 (mTORC1) signaling, and rapid increases in synaptic protein synthesis that underlie new synapse formation. Negative allosteric modulators, including CP-101,606, CERC-301, and Ro 25-6981 agents like rapastinel, may increase synapse formation by enhancing NMDA function and thereby increasing BDNF release and downstream mTORC1 signaling. A role for mTORC1 is further supported by evidence that an agent that increases mTORC1 activity also produces synaptic and rapid antidepressant responses. In addition to these sites, there is evidence that the GABA _A_-positive allosteric modulating agents brexanolone and SAGE-217 also produce rapid antidepressant responses. The intersection of these agents with the mechanisms underlying the rapid response to glutamatergic agents remains to be identified.

Glutamate and NMDA receptors play an important role in cellular models of learning and memory, notably long-term potentiation (LTP), characterized by sustained synaptic strengthening in response to prior high-frequency stimulation. Actions of ketamine at NMDA receptors could influence NMDA function and synaptic plasticity in brain regions implicated in depression ^14^. The possibility that synaptic changes are relevant to depression is supported by evidence that chronic stress, often used in rodent models of depression, causes significant loss of synapses and even retraction of apical dendrites in the prefrontal cortex (PFC) and hippocampus ^15, 16^ ( Figure 1). The relevance of stress-induced synaptic deficits in rodent models is in turn supported by evidence from brain imaging studies demonstrating decreased volume of PFC and hippocampus in depressed patients and post-mortem studies showing decreased synapse number ^17^. In contrast to the effects of chronic stress, we found that a single dose of ketamine rapidly increases the number and function of spine synapses in layer V pyramidal neurons in the medial PFC (mPFC) and rapidly reverses the synaptic deficits of these neurons caused by 3 weeks of chronic stress exposure ^18, 19^ ( Figure 1). Increased levels of synaptic proteins, including levels of glutamate AMPA receptor GluA1, were observed after 2 hours, consistent with the onset of the therapeutic actions of ketamine. These studies demonstrate that ketamine rapidly increases synaptic function in the mPFC and that this reverses the synaptic pathophysiology of depression ^20^.

In addition to the effects of ketamine in the mPFC, there is a recent high-impact report demonstrating the effects of ketamine in the lateral habenula, a region that inhibits the major reward centers in the brain ^21^. This study shows that depressive-like behavior in rodent models (rat congenital learned helplessness [LH] and mouse chronic restraint stress) is characterized by increased burst firing of neurons in the lateral habenula, which causes inhibition of the activity of the major reward and emotion pathways and the ventral tegmental dopamine system as well as the serotonin dorsal raphe neurons. This burst firing is driven by NMDA receptor activity as well as low-voltage-sensitive T-type calcium channels; ketamine, after either systemic or local intracerebral administration, is sufficient to block the burst firing and depressive behavior in the LH rats. It has been postulated that blockade of the anti-reward effects exerted by the lateral habenula could underlie the rapid antidepressant actions of ketamine and that the synaptic effects of ketamine in the mPFC could be more related to the sustained actions of ketamine, although further studies are needed to test this hypothesis.

### Mechanisms underlying the synaptic actions of ketamine: disinhibition hypothesis

How might ketamine, an NMDA receptor channel blocker, cause a rapid increase in the number and function of spine synapses? There are different theories, but the one that has received the most attention is that ketamine actually increases glutamate transmission and causes an LTP-like enhancement of synapse formation in the mPFC ^14^. This idea was first formulated when it was discovered that ketamine rapidly (30 minutes after systemic administration) increases extracellular glutamate in the mPFC of rodents ^22^; this effect was observed only at low subanesthetic doses of ketamine, whereas high anesthetic doses had no effect. Elevation of extracellular glutamate led to the hypothesis that low doses of ketamine selectively block NMDA receptors on GABA interneurons that inhibit glutamate transmission ( Figure 1). This selectivity is based on the fact that GABA interneurons are tonic firing, which results in removal of the Mg ^2+^ block of the NMDA receptor channel, allowing ketamine to enter, bind, and block the channel. Direct evidence for the disinhibition hypothesis has been reported recently in slice studies of hippocampus, which show that low concentrations of ketamine decrease inhibitory input onto pyramidal neurons and thereby lead to an increase in the synaptic drive of excitatory pyramidal neurons in the hippocampus ^23^. There is also an opposing “direct” hypothesis that the synaptic actions of ketamine are mediated by blockade of NMDA receptors on excitatory neurons, which leads to homeostatic control of synaptic activity ^24^. This homeostatic direct hypothesis is difficult to rationalize in the mPFC where there is a glutamate burst but could occur in other brain regions where there is no increase in glutamate.

Studies of the cellular signaling mechanisms underlying the synaptic actions of ketamine demonstrate a requirement for brain-derived neurotrophic factor (BDNF) and activation of pathways that increase the synthesis of synaptic proteins ( Figure 1). The antidepressant actions of ketamine are blocked in conditional BDNF-knockout mice ^24^ and in mice with a knockin of the BDNF Val66Met allele, which blocks the processing- and activity-dependent release of BDNF ^25^. Further evidence that activity-dependent release of BDNF is required comes from studies demonstrating that infusion of a function-blocking antibody into the mPFC blocks the antidepressant actions of ketamine ^26^. Increased synthesis of synaptic proteins occurs via regulation of the mechanistic target of rapamycin complex 1 (mTORC1) as well as eukaryotic elongation factor 2 kinase (eEF2K). We have found that ketamine rapidly increases the phosphorylation of mTOR and downstream signaling proteins that stimulate synaptic protein synthesis and that the behavioral actions of ketamine are blocked by infusion of rapamycin, a selective inhibitor of mTORC1 ^18^ ( Figure 1). Further evidence for mTORC1 is provided by recent studies demonstrating that direct stimulation of mTORC1 also produces rapid synaptic and antidepressant behavioral responses ^27^. Ketamine stimulation of mTORC1 signaling has been replicated in multiple laboratories ^18, 19, 28– 42^, although there is a preliminary report of non-significant effects, raising a concern about the suitability of this target for drug screening ^43^. An alternative hypothesis is that ketamine blockade of NMDA receptors at rest results in deactivation of eEF2K and de-suppression of translation, thereby increasing BDNF expression in pyramidal neurons ^24^. There has also been evidence that ketamine influences the opioid system and inflammation processes, effects that could contribute to the actions of ketamine ^44, 45^.

## Development of novel glutamate/NMDA receptor agents for depression

**Figure 2.**
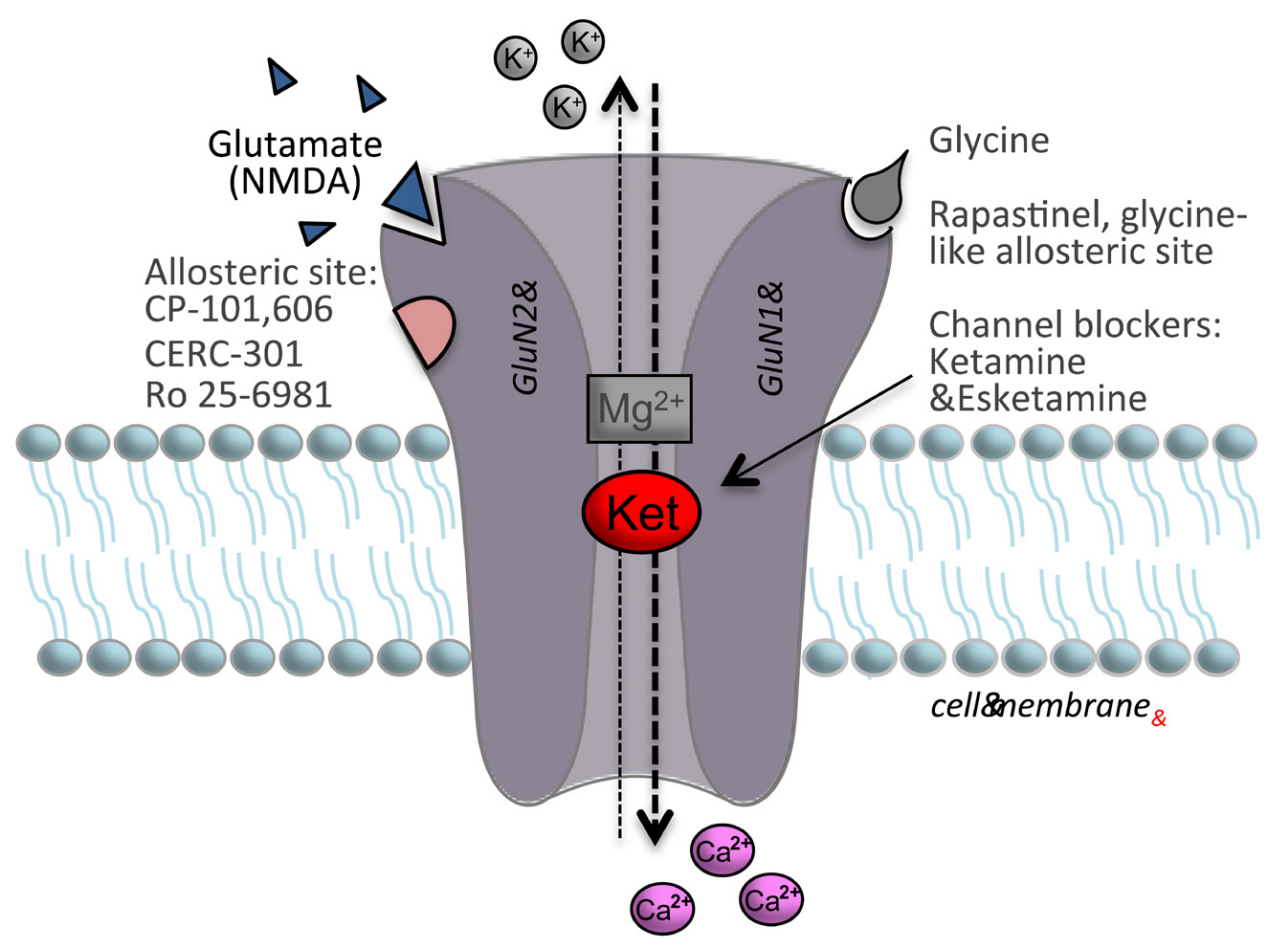
Model of the NMDA receptor complex and target sites of rapid-acting antidepressants. The NMDA receptor is a complex of four subunits comprising four subunits that form a pore that is permeable to Ca ^2+^. At resting state, the pore is blocked by Mg ^2+^, but, upon depolarization, Mg ^2+^ is removed, allowing entry of Ca ^2+^. In the open state, the pore is also accessible by ketamine, which enters and blocks further Ca ^2+^ influx. The (S)-enantiomer esketamine binds to the same site to block the channel. There are several known sites for regulation of the NMDA receptor in addition to the ketamine and glutamate/NMDA binding sites. Glycine binds to the GluN1 subunit and enhances NMDA receptor function; AV-101 is an antagonist of the glycine B co-agonist site. Rapastinel has glycine-like enhancing properties, although it binds to an allosteric site on the complex. There are several GluN2B-selective allosteric modulators that have potential as rapid-acting agents, including CP-101,606, CERC-301, and Ro 25-6981. It is currently unknown what the initial target is for the metabolite (2R,6R)-hydroxynorketamine.

The widespread use of ketamine for the treatment of depression is limited by its psychotomimetic and dissociative side effects as well as abuse potential. Nevertheless, for treatment-resistant patients, ketamine may be the only choice and is becoming more widely available. Another limitation is that ketamine is administered primarily by an IV route in a clinic or hospital setting. To address this issue, Johnson & Johnson is developing a nasal application of the (S)-ketamine stereoisomer (esketamine), which has received breakthrough therapy classification from the US Food and Drug Administration (FDA). Esketamine acts similarly to ketamine and has higher affinity as an NMDA channel blocker than the (R)-isomer ( Figure 2). Early clinical trials have been promising, and although esketamine has side effects similar to those of ketamine, it could be approved as a nasal application as early as the beginning of 2019. Surprisingly, clinical trials with other non-selective NMDA receptor antagonists, including memantine and lanicemine, have been largely negative ^46– 48^. The reason for the lack of efficacy is not clear but could be related to the dose used or the efficacy (or both) of the channel-blocking activity of these agents.

### Stereoisomers and metabolites of ketamine

Efforts have also continued to develop and identify agents with the rapid and efficacious actions of ketamine but with fewer or no side effects. There is evidence from rodent studies that the (R)-stereoisomer of ketamine also produces rapid antidepressant effects in rodent models but without the ketamine-like side effects on sensory motor gating or conditioned place preference ^49^. The reduced side effects may be related to the decreased affinity of the (R)-isomer for the NMDA receptor. In addition, (S)- but not (R)-ketamine is reported to stimulate mTORC1 signaling ^42^. There is also evidence that the ketamine metabolite (2R,6R)-hydroxynorketamine—(2R,6R)-HNK—produces rapid and sustained antidepressant actions in several rodent models, again without influencing sensory motor gating or conditioned place preference ^50^. This study found that (2R,6R)-HNK increased AMPA-GluA1 receptor expression and activity and increased levels of BDNF and phosphorylation of eEF2K; we have also found that the behavioral actions of (2R,6R)-HNK are blocked in BDNF Val66Met mice or by infusion of a function-blocking antibody and by infusion of rapamycin into the mPFC ^51^. However, others have reported that the antidepressant actions of (2R,6R)-HNK are less potent and stable compared with (R)-ketamine ^52^ or do not produce antidepressant actions in a rat LH model ^53^. Zanos and colleagues ^50^ report that they could find no evidence that (2R,6R)-HNK blocks NMDA receptors, including binding to known sites on the receptor complex or NMDA activity in hippocampal slices, so the initial cellular trigger remains to be identified. Nevertheless, another study reported that a high concentration of (2R,6R)-HNK (50 µM) produces partial blockade of synaptic NMDA receptors and downstream signaling compared with the same concentration of ketamine ^54^.

### Other glutamatergic agents: GluN2B and mGluR2/3 antagonists and rapastinel

There is also evidence from rodent studies and preliminary clinical trials that antagonists that are selective for the GluN2B subunit of the NMDA receptor have antidepressant efficacy ( Figure 2). Clinical studies first reported that a single dose of the selective GluN2B antagonist CP101,606 produces antidepressant actions in depressed patients, although significant effects were not observed until 5 days later ^55^. Recent clinical studies with another GluN2B-selective agent, CERC-301, have been mixed; early studies reported positive effects, and a more recent report was negative, although this could be because of the lower dose used for the latter study ^56^. Rodent studies demonstrate that a single dose of the GluN2B antagonist Ro 25-6981 produces antidepressant actions in several different antidepressant models, including the forced swim test (FST), LH, and novelty suppressed feeding test (NSFT), as well as in the chronic unpredictable stress-anhedonia model ^18, 57^. Additional studies are needed to further test the efficacy and rapid actions of GluN2B-selective agents.

Another agent of interest is rapastinel, also referred to as GLYX-13, which was initially thought to act as an NMDA glycine site partial agonist ( Figure 2). More recent studies indicate that rapastinel has activity that is similar to that of a glycine site partial agonist but acts via an allosteric site ^58^. Preclinical studies demonstrate that a single dose of rapastinel results in antidepressant actions in several rodent models, including the FST, NSFT, LH, chronic mild stress/anhedonia, and social defeat stress ^27, 59^. In contrast to ketamine, rapastinel does not influence sensory motor gating or conditioned place preference. A single dose of rapastinel also increases synapse number and function in the mPFC and requires BDNF release and mTORC1 activation ^27, 41^. However, one study reports that the antidepressant actions of rapastinel in a social defeat model as well as its effects on BDNF-TrkB signaling are not as long-lasting as those of (R)-ketamine ^60^. A double-blind randomized clinical trial reported that a single dose of rapastinel produces antidepressant actions that persisted for about 7 days ^61^. Rapastinel has received breakthrough classification and currently is in phase III clinical trials. Another agent that works via the glycine site is AV-101, a prodrug (L-4-chlorokynurenine) that is transported into the brain where it is converted to 7-chlorokynurenic acid, a potent antagonist of the glycine B coagonist site of the NMDA receptor ^62^; AV-101 is in phase II clinical trials and has received fast-track status from the FDA.

Based on evidence that the actions of ketamine occur via a “glutamate burst” and the subsequent enhancement of synaptic function, other approaches to increase glutamate neurotransmission have been tested. Most notable are the metabotropic GluR2/3 antagonists, which increase glutamate activity by blocking presynaptic autoreceptors ( Figure 1). Preclinical studies have reported that mGluR2/3 antagonists, notably LY341,495 and MGS0039, produce antidepressant effects in a number of rodent models, including the FST, NSFT, and a chronic unpredictable stress-anhedonia model, and also require mTORC1 signaling ^29, 63, 64^. Clinical trials are needed to determine whether blockade of mGluR2/3 receptors produces a therapeutic response in patients and to determine the side effect profile of these agents.

## Additional rapid-acting antidepressant approaches

Although drug development has focused on direct-acting glutamatergic approaches, there is evidence for other initial targets that produce rapid antidepressant responses, although some of these also indirectly influence glutamate transmission. The most notable of these is scopolamine, a non-selective acetylcholine muscarinic (AChM) receptor antagonist, which has shown promise in clinical trials. These studies have reported that a single low dose (4 µg/kg IV) of scopolamine produces an antidepressant response at the first assessment time point, 3 days after dosing, and there is anecdotal evidence of improvement within 24 hours ^65, 66^. These rapid therapeutic actions led us to examine the cellular actions of scopolamine in rodent models. These studies showed that, like ketamine, scopolamine causes a rapid burst of glutamate in the mPFC and increases mTORC1 signaling and synapse formation and that the antidepressant actions of scopolamine require BDNF release and mTORC1 ^67, 68^ ( Figure 1). We have also found that the antidepressant actions of scopolamine occur through blockade of AChM1 receptors on GABA interneurons, demonstrating that scopolamine acts via a disinhibition mechanism ^69^. Despite these interesting findings, there has been less interest in developing AChM antagonists for the treatment of depression relative to ketamine and direct-acting glutamatergic agents.

Another indirect approach that influences glutamate transmission is via the regulation of GABA _A_ receptors, particularly selective inverse agonists that would increase glutamate transmission. Rodent studies targeting the α5-subunit-containing GABA _A_ receptor, which is enriched in the PFC and hippocampus, report that a single dose of the α5-selective inverse agonists L-655,708 or MRK-016 produce rapid antidepressant behavioral responses in a number of tests, including the FST and female urine sniffing test (FUST), and chronic restraint stress and chronic unpredictable stress-anhedonia models ^70, 71^. The actions of these inverse agonists require AMPA receptor activity, indicating an increase in glutamate transmission as expected. These compounds lack the side effects of ketamine on sensory motor gating and conditioned place preference, and this is possibly due to the restricted expression of α5-subunit-containing GABA _A_ receptors. Clinical studies are required to determine the therapeutic potential and to confirm the reduced side effect profile of these agents.

One other very different approach is based on evidence that ketamine and several other rapid-acting agents increase mTORC1 signaling in the mPFC. The biotech company Navitor has developed an agent, NV-5138, that stimulates mTORC1 signaling via binding to sestrin, an upstream regulatory pathway ^72^ ( Figure 1). We have found that a single oral dose of NV-5138 increases mTORC1 signaling in the mPFC as expected and produces rapid antidepressant actions in the FST, NSFT, FUST, and chronic unpredictable stress-anhedonia models ^27^. These behavioral actions of NV-5138 are long-lasting (up to 7 days). In addition, a single dose of NV-5138 increases synapse number and function and levels of synaptic proteins in the mPFC. Preliminary studies also demonstrate that the actions of NV-5138 require BDNF (unpublished). Based on these promising preclinical findings, efforts are under way to test NV-5138 in human subjects and depressed patients.

Another exciting finding is the recent report that the neuroactive steroid allopregnanolone (SAGE-547, now referred to as brexanolone) produces rapid therapeutic actions in women with post-partum depression (PPD) ( Figure 1). Allopregnanolone is a positive allosteric modulator of synaptic and extrasynaptic GABA _A_ receptors, particularly the δ-subunit that regulates tonic firing of GABA interneurons. PPD is associated with the precipitous drop at birth of allopregnanolone and other progesterone-derived neurosteroids that are very high during pregnancy; since PPD occurs in a subpopulation of women, there are likely vulnerability factors that are also involved. Previous studies have reported that mice with a deletion of the δ-subunit show depression-like behavior and abnormal maternal care during the post-partum period ^73^. The clinical study reports that sustained infusion (IV) of brexanolone results in a therapeutic response within 24–48 hours in severely depressed PPD women ^74, 75^. Brexanolone has received breakthrough status for the treatment of PPD and currently is in phase III trials. In addition, Sage has developed an orally available synthetic analogue, SAGE-217, that has shown therapeutic efficacy in both female and male patients with major depression; SAGE-217 has also received breakthrough therapy status and soon will be starting phase III trials.

## Future studies and drug development

The discovery of ketamine and related agents holds tremendous promise for the rapid, efficacious treatment of depression. We are experiencing an unprecedented time of antidepressant drug development in this area; at least five different agents (rapastinel, esketamine, AV-101, brexanolone, and SAGE-217) have been awarded breakthrough therapy or fast-track status from the FDA. However, there are several critical problems to overcome. First, ketamine is a drug of abuse and has serious side effects, so new agents with a reduced side effect profile are needed. Second, although ketamine produces a rapid antidepressant response, the effects last for about 1 week, at which time patients typically relapse. New agents that can be used on a daily, sustained basis are needed. Third, studies are needed to understand why new ketamine-induced synapses are lost after 1 week and whether there are approaches or agents (or both) that can sustain the synaptic as well as the therapeutic actions of ketamine. Fourth, additional research is needed to fully understand the cellular mechanisms underlying the actions of ketamine and other rapid-acting agents and to determine the critical pathophysiological abnormalities that cause depression. The future for novel rapid-acting antidepressants looks very bright, as the drugs currently being tested in phase II and III clinical trials may address some of these issues. With continued efforts, there is hope that there will soon be a number of novel, rapid, and efficacious choices for the treatment of depression and the possibility that these could target the underlying causes of illness.
